# Comparison of pre- and intra-COVID-19 postpartum depression among reproductive aged women: A comparative cross-sectional study in Ahvaz, Iran

**DOI:** 10.3389/fpsyt.2022.1019432

**Published:** 2022-11-08

**Authors:** Poorandokht Afshari, Mitra Tadayon, Parvin Abedi, Maryam Beheshtinasab

**Affiliations:** ^1^Midwifery Department, Reproductive Health Promotion Research Center, Ahvaz Jundishapur University of Medical Sciences, Ahvaz, Iran; ^2^Midwifery Department, Menopause Andropause Research Center, Ahvaz Jundishapur University of Medical Sciences, Ahvaz, Iran

**Keywords:** postpartum depression, COVID-19 pandemic, reproductive aged women, cross-sectional study, postnatal depression

## Abstract

**Background:**

The association between PPD and COVID-19 pandemic has been studied in some countries. This study aimed to compare postpartum depression before and during the COVID-19 pandemic among reproductive-aged women in Ahvaz, Iran.

**Methods:**

This comparative cross-sectional study involved 600 women of reproductive age in Ahvaz, Iran during the COVID-19 pandemic who were compared with 504 of their counterparts before the pandemic. Literate women who had given birth 1–6 months prior to the study, were aged 18–35, and were willing to participate in this study were recruited. A demographic questionnaire and Edinburgh Postnatal Depression Scale were used to collect the data. Independent *t*-test, Chi-square, and Logistic regression were used to analyze the data.

**Results:**

Before the pandemic, only 123 (24.9%) of the women had PPD, while during the pandemic, this number rose to 409 (68.2%). During the COVID-19 pandemic, the women were 8.727 times more likely to have PPD (95% CI: 5.919–12.868). During the pandemic, women with high school education and those having a high school diploma were 2.454 and 2.054 times more likely to have PPD compared to women with a university degree (95% CI: 1.508–3.996 and 1.302–3.241, respectively).

**Conclusion:**

The prevalence of PPD among Iranian reproductive-aged women increased during the COVID-19 pandemic. Policymakers should seek some strategies to support women during pregnancy and postpartum in times of crises such as the COVID-19 pandemic.

## Introduction

Postpartum depression (PPD) is a severe but treatable disorder that includes extreme sadness, anxiety, and changes in level of energy, sleep and appetite, and it occurs in some women within 4 weeks after giving birth (1). Unfortunately, postpartum depression is on the rise in all societies. For example, in a systematic review on 565 studies, Wang et al. (2) found that the prevalence of PPD is 17.22%. They found high heterogeneity due to the developed or underdeveloped countries under study. Iran as a developing country has been found to have high prevalence of PPD. According to a systematic review including 1,165 participants by Veisani and Sayehmiri (3), the prevalence of PPD was 28.7% among Iranian women, and this rate was 39.6% in women with a positive history of depression. A recent study in Khuzestan, Iran showed that the prevalence of PPD is 38.8%, and factors such as history of PPD, having neonates with congenital malformation, and admission of neonates to intensive care unit were effective predictors of PPD (4).

PPD has maternal and neonatal consequences. Problems related to physical health, psychological and communication problems, and dangerous behaviors are examples of maternal consequences of PPD, while decreased neonatal anthropometric measures, sleep disturbances, and motor and cognitive retardation are the negative consequences of PPD affecting neonates and infants (5). Albeit not very common, suicidal ideation is one of the consequences of PPD in women which is a source of concern and worry (6).

The association between PPD and COVID-19 pandemic has been studied in some countries. In a study conducted in the United States, for example, Shuman et al. (7) found that one-third and one-fifth of the studied women had PPD and severe PPD, respectively. Also, in a systematic review by Chen et al. (8) found that the prevalence of PPD during COVID-19 pandemic was 34%, which was much higher than the same rate before emergence of the pandemic. Another systematic review by Safi-Keykaleh et al. (9) which included 24 studies found that according to Edinburgh Postnatal Depression Scale (EPDS) and considering score >13, the prevalence of PPD during COVID-19 pandemic was 28%. However, there is evidence suggesting no difference between PPD before and after the pandemic. For example, Waschmann et al. (10) in their study on 557 women before pandemic and 504 women after pandemic found that the prevalence of PPD was similar in the two groups.

Although a number of studies have been conducted on PPD before emergence of the COVID-19 pandemic in Iran, there is paucity of research regarding the prevalence of and factors associated with PPD during this pandemic in Iran. Therefore, this study was designed to compare the prevalence of PPD and the factors affecting it before and after the COVID-19 pandemic in Ahvaz, Iran.

## Materials and methods

This was a comparative cross-sectional study conducted on 600 women in reproductive age in Ahvaz, Iran. The data of this study was compared to that of 504 women in reproductive age obtained before emergence of the COVID-19 pandemic. This study started in August 2021 and completed in January 2022 (which was concurrent with the fifth surge of COVID-19 in Iran). The protocol of this study was approved by the Ethics Committee of Ahvaz Jundishapur University of Medical Sciences, Ahvaz, Iran (Ref No: IR.AJUMS.REC.1400.246).

### Inclusion/exclusion criteria

Women with basic literacy who had given birth 1–6 months prior to the study, were aged 18–35 and resident in the urban areas, and had willingness to participate in this study were recruited. Women with physical or mental disorders during pregnancy, and those experiencing stressful events during the 6 months prior to the study or having a disabled child were excluded from the study. The inclusion/exclusion criteria for postpartum women before pandemic was similar to those of the present study (4).

### Sampling

Six public health centers (three from the west bank and three from the east bank of the Karun River in Ahvaz, Iran) were chosen randomly from among 60 centers. The following formula was used for sampling:


$$\begin{array}{l} n=\frac{z_{1-\alpha /2}^{2}\times p\left(1-p\right)}{d^{2}} \end{array}$$

A total number of 600 was considered for sampling. We enrolled 100 women from each public health center. One of the researchers (MB) visited each of these public health centers on a daily basis and prepared a list of women who had given birth to be later contacted. Eligible women who provided written informed consent were requested to fill-up two questionnaires, and the same research team member (MB) was available in case the participants had any question.

### Measures

A demographic questionnaire and Edinburgh Postnatal Depression Scale (EPDS) were used to collect data. The demographic questionnaire included questions about age, occupation, educational attainment, number of children, economic status, and husband's age and educational attainment. The content validity of this questionnaire was approved.

EPDS has 10 questions and assesses the feelings of respondents during the past 7 days. The scores of each question ranges from zero to three. The total score of this questionnaire is 30, and scores more than 10 represent postnatal depression (11). The validity and reliability of this questionnaire were approved by Mazhari and Nakhaee (12) and Galini Moghadam et al. (13) in Iran. EPDS was also the same tool used for assessing PPD before pandemic in 2019 (4).

### Procedure

Eligible women were invited to each center and asked to complete the demographic and EPDS questionnaires. If they had difficulty in completing the questionnaire, one of the researchers (MB) was available to help them. A group of 504 women who were assessed in 2019 for the prevalence of postpartum depression and the factors affecting it were considered as a control group (4). To abide by ethical considerations, after analyzing the data, those women who were screened with EPDS and scored higher than 10, were recommended to be visited by a psychiatrist.

### Statistical analysis

Data of this study was analyzed using SPSS version 22. The Shapiro–Wilk test was used to test the normal distribution of data. Independent *t*-test and Chi-square tests were used to test differences between the two groups regarding continuous and categorical data, respectively. Logistic regression was used to test differences between the two groups regarding depression with a 95% confidence interval. *P* < 0.05 was considered statistically significant.

## Results

In this study, 850 women in postpartum period were screened of whom 600 were found to be eligible and were compared with 504 of their counterparts before the pandemic. Demographic characteristics of the participants during the COVID-19 pandemic are illustrated in Table 1. The mean ± SD age of women was 28.46 ± 6.38 and 29.07 ± 6.3 in women without and with PPD, respectively. The two groups did not show any significant difference regarding husband's age, number of children, educational attainment of the participants and their husbands, and the participants' and their husband's occupation. Women without PPD had significantly better economic status in comparison with women with PPD (*p* = 0.02).

**Table 1:** Demographic characteristics of participants according to the severity of depression during COVID-19 pandemic.

**Variables**	**Normal** ***n* = 191**	**Postpartum depression** ***n* = 409**	***P-*value**
	**Mean** ±**SD**		
Age (y)	28.46 ± 6.38	29.07 ± 6.3	0.272
Husband's age (y)	30.02 ± 6.8	30.57 ± 6.04	0.313
	***n*** **(%)**		
**Number of children**		
1	79 (41.3)	157 (38.3)	0.634
2–3	104 (54.4)	229 (55.9)	
>4	8 (4.18)	23 (5.6)	
**Education**		
High school	22 (11.5)	49 (11.9)	0.988
Diploma	24 (12.5)	50 (12.2)	
University	145 (75.9)	310 (75.7)	
**Occupation**		
Housewife	93 (48.6)	201 (49.1)	0.517
Employee	98 (51.3)	208 (50.8)	
**Husband's education**		
High school	42 (21.9)	92 (22.4)	0.786
Diploma	51 (26.7)	99 (24.2)	
University	98 (51.3)	218 (53.3)	
**Husband's occupation**		
Unemployed	22 (11.5)	51 (12.4)	0.408
Government employee	113 (59.1)	264 (64.5)	
Self-employed	56 (29.3)	95 (23.2)	
**Economic status**		
Good	35 (18.3)	53 (12.9)	0.024
Moderate	130 (68.06)	266 (65.03)	
Poor	26 (13.6)	90 (22)	
History of PPD*		
Yes	19 (9.9)	39 (9.5)	0.774
No	93 (48.6)	213 (52)	
Primipara	79 (41.3)	157 (38.3)	
**Medical disorders in pregnancy**		
Yes	16 (8.37)	87 (21.2)	< 0.0001
No	175 (91.6)	322 (78.7)	

The * symbol indicates the postpartum depression.

There was no significant difference between the two groups regarding history of PPD. Women with PPD had significantly more medical disorders during their pregnancy (*p* < 0.0001).

Factors related to COVID-19 infection among women with and without PPD during the COVID-19 pandemic were assessed. There was no significant difference between the two groups regarding the effect of pandemic on the decision for conception, having COVID-19 infection during current pregnancy, and hospitalization due to infection. Surprisingly, the rate of vaccination among women without PPD was significantly lower in comparison to women with PPD (*p* = 0.011) (data are not shown in table).

When we pooled data for women with and without PPD before and after COVID-19 pandemic, women with PPD were significantly older, were less employed, and had higher university education (*p* < 0.05; Table 2). Before the pandemic, 191 (31.8%) of the women had PPD, while this rate rose to 409 (68.2%) during the pandemic (*p* = 0.0001).

**Table 2:** Comparison of women with and without PPD before and after COVID-19 pandemic.

**Variables**	**Normal** ***n* = 572**	**Postpartum depression** ***n* = 532**	***P-*value**
	**Mean** ±**SD or** ***n*** **(%)**		
Women's age (y)	27.5 ± 5.68	28.78 ± 6.0	0.001
**Occupation**
Housewife	390 (68.18)	219 (41.16)	0.0001
Employee	253 (44.23)	243 (45.6)	
**Education**
High school	108 (18.8)	89 (16.7)	0.007
Diploma	142 (24.8)	99 (18.6)	
University degree	316 (55.2)	351 (65.9)	

For a better understanding of the relationship of depression with COVID-19 pandemic we used logistic regression, and the results of this test are presented in Table 3. As this table shows, postpartum women during COVID-19 pandemic were 8.727 times more likely to have PPD (95% CI: 5.919–12.868). Also, postpartum women with high school education and those having a high school diploma were 2.454 and 2.054 times more likely to have PPD compared to women with a university degree (95% CI: 1.508–3.996 and 1.302–3.241, respectively).

**Table 3:** Logistic regression of the relationship between postpartum depression and COVID-19 pandemic.

**Variables**	**β**	**S.E**.	**Wald**	**df**	**Sig**.	**Exp (β)**	**95% CI for Exp (**β**)**	
							**Lower**	**Upper**
Pandemic	2.166	0.198	119.585	1	0.000	8.727	5.919	12.868
Job	−0.177	0.185	0.920	1	0.338	0.838	0.583	1.203
Age	0.018	0.014	1.663	1	0.197	1.019	0.991	1.047
Education			15.440	2	0.000			
Under diploma	0.898	0.249	13.039	1	0.000	2.454	1.508	3.996
Diploma	0.720	0.233	9.567	1	0.002	2.054	1.302	3.241
Constant	−1.985	0.442	20.193	1	0.000	0.137		

## Discussion

This study was designed to evaluate the relationship between the COVID-19 pandemic and PPD. Our results showed that COVID-19 pandemic was a risk factor for PPD. During the pandemic, 68.2% of the women had PPD compared to 31.8% before pandemic, which is consistent with previous studies. Shuman et al. (7), for example, in a study on 670 American women in postpartum period found that one out of three women participating in their study had PPD, and one in five women had severe depression, while before the pandemic this rate was 6.5–12.9%. Contrary to our results, however, Erten et al. (14) in a study in Turkey found that the prevalence of PPD was 17.4%, which is much smaller than what we found in our study. This discrepancy may be attributed to the fact that we collected data when vaccination of pregnant women had not become a routine practice in Iran. In fact, 32.4 and 21.2% of the women without and with PPD in our study had not received vaccination and around 40% of the women in the two groups had received only one dose of vaccine. Furthermore, during home quarantine in Iran, pregnant women might not have received sufficient care from health providers, or they may have refused to attend the clinics (15). Also, prenatal and postnatal visits during this period were so short that evaluation of mental health was not possible.

In a systematic review including 6,480 postpartum women mostly were from developed countries, Chen et al. (8) found that the prevalence of PPD was 34% during the pandemic, which was much higher than the rate reported before the pandemic. This prevalence is much lower than what we found in our study (68.2% of women had PPD). This discrepancy could be explained by the fact that the prevalence of PPD among Iranian women before the COVID-19 pandemic was higher than that in developed countries, as attested by Veisani and Sayehmiri's (3) who reported a prevalence of 28.7% for PPD in a systematic review.

Our results showed that COVID-19 pandemic increased the risk of PPD by 8.727. In a study on 330 Greek women in postpartum period, Micha et al. (16) found that while the prevalence of PPD was 13.2%, many of the studied women (24.8%) showed high levels of antenatal anxiety during the COVID-19 pandemic. According to Nakic Rados et al. (17), the rate of anxiety during pregnancy is high, which reduces immediately after childbirth and then slightly increases 6 weeks postpartum. They found high rate of comorbidities (75%) between anxiety and PPD. Unfortunately, in the current study we did not measure the participants' level of anxiety and stress since the high level of anxiety during COVID-19 pandemic and lack of sufficient vaccination might have been predisposing factors for PPD.

Our results showed that women with high school education and those having a high school diploma were 2.454 and 2.054 times more likely to have PPD compared to women with university degrees. Also, Liang et al. (18) reported that 30% of the studied women had PPD, and factors such as: immigration, poor social support, and worry about getting COVID-19 infection were associated with PPD. It is worth mentioning that the factors associated with PPD are different from one society to another.

### Strengths and limitations of the study

This was the first study to evaluate the postpartum depression of Iranian women during COVID-19 pandemic. For a better understanding of the effect of the pandemic, we compared our results with a previous study that was conducted in 2019 before the pandemic. Despite its strengths, however, this study has some limitations. First we did not recruit women randomly, and this may affect the generalizability of the findings. Second, we did not check the anxiety, fear, or stress of women along with their depression. According to other studies, anxiety and stress may trigger PPD.

## Conclusion

The results of this study showed that the prevalence of PPD was doubled during the COVID-19 pandemic. Therefore, policymakers are recommended to consider some strategies to support women during pregnancy and postpartum period especially during crises such as COVID-19 pandemic. Further studies measuring anxiety, stress, and PPD are needed to draw a definite conclusion about PPD during crises such as COVID-19 pandemic.

## Data availability statement

The raw data supporting the conclusions of this article will be made available by the authors, without undue reservation.

## Ethics statement

The studies involving human participants were reviewed and approved by Ahvaz Jundishapur University of Medical Sciences. The patients/participants provided their written informed consent to participate in this study.

## Author contributions

Material preparation and data collection were performed by MB. Data analysis and interpretations were done by PAb and PAf. The first draft of the manuscript was written by PAb. All authors commented on the earlier versions of the manuscript, contributed to the study conception and design, read, and approved the final manuscript.

## Funding

All expenses of this study were provided by Ahvaz Jundishapur University of Medical Sciences. The funder did not have any role in design, data collection, analysis and interpretation, and writing and submitting the manuscript to a journal.

## Conflict of interest

The authors declare that the research was conducted in the absence of any commercial or financial relationships that could be construed as a potential conflict of interest.

## Publisher's note

All claims expressed in this article are solely those of the authors and do not necessarily represent those of their affiliated organizations, or those of the publisher, the editors and the reviewers. Any product that may be evaluated in this article, or claim that may be made by its manufacturer, is not guaranteed or endorsed by the publisher.
